# 

**Published:** 1792

**Authors:** 


### CONTENTS.

Page

I. *CASE of a compound Fracture of the Leg; with Remarks. By Mr.* Henry Yates Carter, *Surgeon at Kettley, near Wellington, in Shropshire.* — — 1

II. *Case of a Boy whose Head was pressed between certain Parts of an Engine employed for draining a Coal Mine. By the Same.* 11

III. *Case of a Boy whose left Leg and Thigh, together with Part of the Scrotum, were torn off by a Slitting Mill. By the Same.* 17

IV. *Case of a fungous Enlargement of the Extremity of the female Urethra; with Remarks. By Mr.* T. Hughes, *Surgeon at Stroud Water in Gloucestershire.* — 26

V. *Case of Emphysema, brought on by severe Labour Pains. By Mr.* R. B. Blagden, *Surgeon at Petworth in Sussex.* — 45

VI. *An Account of the spontaneous Cure of an Aneurism. By the Same.* — 48

VII. *Some*

Page

VII. *Some Remarks on the Anguſtura Bark. By Mr.* George Wilkinſon, *Surgeon at Sunderland, Member of the Royal College of Surgeons and honorary Member of the Chirurgo-phyſical Society of Edinburgh,* &c. 52

VIII. *An Account of two Caſes of Polydipſia, or exceſſive Thirſt.* — — — 73

IX. *An Account of the good Effects of Electricity in a Caſe of paralytic Affection; ſerving to prove that, in ſuch Caſes, the electric Sparks ſhould be taken from the Muſcles which are Antagoniſts to thoſe that are contracted. By* William Gilby, *M. D. Phyſician to the General Hoſpital at Birmingham.* — — — — 102

X. *Obſervations on ſome epidemical Effects. By Mr.* William Blizard, *F. R. S. and S. A. Correſponding Member of the Royal Society of Sciences of Gottingen, and Surgeon to the London Hoſpital.* — 105

XI. *Account of a Method of curing Burns and Scalds. By Mr.* David Cleghorn, *Brewer in Edinburgh.* — — — 120

XII. *An Account of the Cure of a preternatural Anus; with Remarks on the Hiſtory and*

*Treat-*

Page

*Treatment of Cases of this Kind. By M.* Desault, *Surgeon in Chief of the Hotel Dieu at Paris. From the Journal de Chirurgie.* 153

XIII. *Experiments and Observations on the Matter of Cancer, and on the aërial Fluids extricated from animal Substances by Distillation and Putrefaction; together with some Remarks on sulphureous hepatic Air. By* Adair Crawford, *M. D. F. R. S. From the Philosophical Transactions.* — 182

*Catalogue of Books.* — — — 202

*Index.* — — — — 225

DIREC-

